# Heart rate slopes during 6‐min walk test in pulmonary arterial hypertension, other lung diseases, and healthy controls

**DOI:** 10.14814/phy2.12038

**Published:** 2014-06-11

**Authors:** Adriano R. Tonelli, Xiao‐Feng Wang, Laith Alkukhun, Qi Zhang, Raed A. Dweik, Omar A. Minai

**Affiliations:** 1 Department of Pulmonary, Allergy and Critical Care Medicine, Respiratory Institute, Cleveland Clinic, Cleveland, Ohio; 2 Respiratory Institute Biostatistics Core, Quantitative Health Sciences, Cleveland Clinic, Cleveland, Ohio

**Keywords:** Chronic obstructive pulmonary disease, heart rate, pulmonary hypertension, restrictive lung disease, 6‐min walk test

## Abstract

Six‐minute walk test (6MWT) continues to be a useful tool to determine the functional capacity in patients with vascular and other lung diseases; nevertheless, it has a limited ability to predict prognosis in this context. We tested whether the heart rate (HR) acceleration and decay slopes during the 6‐m walk test are different in patients with pulmonary arterial hypertension (PAH), other lung diseases, and healthy controls. In addition, we assessed whether the HR slopes are associated with clinical worsening. Using a portable, signal‐morphology‐based, impedance cardiograph (PhysioFlow Enduro, Paris, France) with real‐time wireless monitoring via a Bluetooth USB adapter we determined beat‐by‐beat HR. We included 50 subjects in this pilot study, 20 with PAH (all on PAH‐specific treatment), 17 with other lung diseases (obstructive [*n *= 12, 71%] or restrictive lung diseases [5, 29%]), and 13 healthy controls. The beat‐by‐beat HR curves were significantly different among all three groups of subjects either during the activity or recovery of the 6MWT. HR curves were less steep in PAH than the other two groups (*P* < 0.001). HR acceleration rates were slower in patients with PAH or other lung diseases with progression of their disease (*P* < 0.001). In conclusion, the acceleration and decay slopes during 6MWT are different among patients with PAH, other lung diseases, and healthy controls. The HR slopes during 6MWT were steeper in patients without clinical worsening.

## Abstract (precis)

For the first time, we studied study the heart rate (HR) acceleration and decay slopes during the 6‐min walk test (6MWT) in patients with pulmonary arterial hypertension (PAH) and compared them to subjects with parenchymal lung diseases and healthy controls. We analyzed a large number (>600) of standardized HR determinations in each patient and applied modern statistical analysis (nonlinear mixed models) to construct the slopes during the activity and recovery phases of the 6MWT. We found that the slopes are less steeper in PAH patients when compared with controls. The slopes of patients with parenchymal lung disease fell in‐between individuals with PAH and healthy controls. More importantly, patients with PAH or parenchymal lung disease has a lower acceleration or deceleration slope if their disease progressed during the follow‐up.

## Introduction

Six‐minute walk test (6MWT) is a useful test to assess the functional status of patients with vascular or other lung diseases. Traditionally, 6MWT has been used to assess disease severity (Humbert et al. 2010; Benza et al. 2012), progression, and response to treatment in patients with pulmonary arterial hypertension (PAH; Badesch et al. 2000; Olschewski et al. 2002; Rubin et al. 2002; Simonneau et al. 2002, 2008; Galie et al. 2009, 2005, 2008; McLaughlin et al. 2010). However, recent publications have questioned its value, since the change in 6‐min walk distance after treatment did not predict the need for PAH hospitalizations, PAH rescue therapy, lung transplantation, or mortality (Macchia et al. 2007; Savarese et al. 2012).

In recent years, investigators have focused on parameters other than the 6‐min walk distance to enhance the information obtained from this simple, safe, and inexpensive examination that is representative of daily life activities. These relatively new determinations include chronotropic response (Provencher et al. 2006), heart rate recovery (HRR; Minai et al. 2012), need for oxygen (Batal et al. 2012), and noninvasive hemodynamic estimations using portable impedance cardiography (Tonelli et al. 2013).

It remains unclear how beat‐to‐beat HR behaves in subjects with PAH during 6MWT and how it compares with individuals with other lung diseases and healthy controls. Therefore, we obtained real‐time beat‐by‐beat HR measurements during 6MWT using a portable impedance cardiograph, namely, Physioflow enduro (Manatec Biomedical, Paris, France). We hypothesized that the slopes of the HR increase and decrease during the activity and recovery phase of the 6MWT will be different among these three groups. We further hypothesized that there will be a difference in the acceleration and decay HR slopes of patients with PAH or other lung diseases who experience disease progression during their follow‐up.

## Methods

This prospective study was approved by the Cleveland Clinic Institutional Review Board (study number 11–226). Written informed consent was obtained from all patients and healthy controls before enrollment. Patients were asked to participate before the 6MWT. The study was performed between August 2011 and November 2012. The 6MWT was ordered by the patient's pulmonary physicians or performed as a part of this research study in healthy controls.

We included patients with PAH confirmed by right heart catheterization (*n *= 20), other lung diseases characterized by abnormal spirometry and imaging lung studies (*n *= 17), and healthy controls matched by age and gender with PAH patients (*n *= 13). We included PAH patients regardless of the PAH‐specific treatment. All patients with other lung diseases had an echocardiogram that revealed an estimate right ventricular systolic pressure of <40 mmHg. Spirometry and the 6MWT were performed on the same day. Healthy controls were recruited by placing recruitment flyers in our outpatient clinic. We excluded patients with atrial fibrillation or paced rhythm. Information about baseline demographics, medication history, clinical, echocardiographic, and hemodynamic determinations were gathered on the day of the 6MWT.

We used a portable (dimensions 11.5 × 8.5 × 1.8 cm, weight of <200 g), new generation, signal‐morphology‐based impedance cardiograph with real‐time wireless monitoring via a Bluetooth USB adapter (PhysioFlow Enduro, Paris, France; Tonelli et al. 2013). A detailed explanation of the methodology used has been previously published (Tonelli et al. 2011, 2013). Physioflow enables continuous digital electrocardiographic recording and measurement of all the RR intervals, which can then be used to calculate the beat‐by‐beat HR (60,000/RR interval in ms). The system allows the insertion of digital markers (by pressing the space bar) that in our study signaled the precise separation of all three phases of the 6MWT (rest, activity, and recovery).

We prepped the skin with a mildly abrasive gel (NuPrep) and 70% isopropyl alcohol. We placed six electrodes (pregelled Ag/AgCl, Skintact model FS‐TB wet‐gel; Leonhard Lang Gmbh, Innsbruck, Austria) at the left base of the neck (*n *= 2), left paraspinal muscles at the level of the xyfoid process (*n *= 2), right upper (*n *= 1), and left lower chest (*n *= 1). These last two electrodes are the ones that capture the electrocardiographic signal. The electrodes were connected to the portable impedance cardiograph via an electrode cable. We then connected the device to a portable computer that had a type 1 Bluetooth USB adapter with external antenna that supported 300 m of wireless transmission (SENA UD100 Bluetooth USB Adapter, Sena Technologies, Inc, Seoul, South Korea).

The 6MWT was performed according to ATS standards (ATS Committee on Proficiency Standards for Clinical Pulmonary Function Laboratories 2002). We obtained the reference standards for the 6‐min walk distance from Enright and Sherrill (1998). Patients remained seated for approximately 10 min before the test (during consenting, electrode placement, and connection of the device). We then acquired real‐time beat‐by‐beat determinations of HR. We also recorded HR determinations averaged every 15 s, which were used to construct Table 2. HR change was obtained from subtracting the maximal HR during 6MWT and the resting HR, just before the initiation of the walk (both averaged values over 15 s). At the end of the 6MWT, the patient entered the recovery phase, when they immediately sat down and their HR (among other parameters) was measured for at least 3 min.

### Statistical analysis

Continuous variables were summarized using mean ± standard deviation (SD) or median and interquartile range when appropriate. To minimize the effects of baseline HR we determined the HR percentage change. The first HR during the activity and recovery was the baseline value for each period, respectively. In <5% of all measurements, we removed HR values that differed more than 15% of the previous HR determination (i.e., extrasystoles or artifacts). We built nonlinear mixed effect models (Lindstrom and Bates 1990) for repeated HR determinations during the activity (6 min) and recovery period (3 min). This model allowed us to describe the nonlinear HR response during activity and recovery, with a fixed effect that represent the global trend across all subjects and random effects to control for the between subject variation. We found that our data were adequately described by a three‐parameter logistic model that included two random effects. The equation used is: 
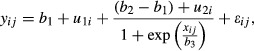


where *i* stands for the number of patients; *j* indicates the repeat time of a patient; *x*_*ij*_ is the elapsed time for each observation, *y*_*ij*_ is the HR value for each observation; *b*_1_, *b*_2_, and *b*_3_, represent the three fixed effects model parameters; *u*_1*i*_ and *u*_2*i*_ are two random effects to account for the random nature of the subject; and *ε*_*ij*_ is the random noise for the model.

We compared the HR curves during activity and recovery among the three groups of patients (PAH, other lung disease, and healthy controls) and we considered them different if any of the three parameters of the logistic model for each phase was statistically different than the comparator. Furthermore, we tested whether there was a difference between the HR curves and between patients with PAH that had a clinical worsening (hospitalized for PAH exacerbation, needed to have a PAH‐specific medication added, underwent lung transplantation, or died) versus the ones that did not. Similarly, we compared the curves between patients with other lung diseases who were hospitalized for respiratory exacerbation or died versus the ones that did not. All patients included in the study were regularly followed in our clinic; therefore, data on outcomes were accurately reflected in our electronic medical records. A *P* value of <0.05 was prespecified as indicative of statistical significance. The statistical analyses were performed using the SAS 9.3 (SAS Institute, Cary, NC, USA) and R studio software (R Project for Statistical Computing, Vienna, Austria).

## Results

### Patient's characteristics

Baseline characteristics are presented in Table 1. Gender was similar among the groups and patients with other lung diseases were older than patients with PAH and controls.

**Table 1.** Patient characteristics

	PAH Mean ± SD or *n* (%)	Other lung diseases Mean ± SD or *n* (%)	Healthy controls Mean ± SD or *n* (%)	*P* (ANOVA/Chi‐square)
*n*	20	17	13	
Age (years)	45.1 ± 12	58.4 ± 11	46.6 ± 10	0.002
Female gender	14 (70)	7 (41)	7 (54)	0.2
Type of disease	Idiopathic:10 (50)	Obstructive:12 (71) Restrictive: 5 (29)		
	Heritable: 3 (15)			
	CHD: 3 (15)			
	CTD: 3 (15)			
	CHA: 1 (5)			
On beta‐blockers	7 (35)	3 (18)		
On CCB	3 (15)	1 (6)		

CCB, calcium channel blockers; CHA, chronic hemolytic anemia; CHD, congenital heart disease; CI, cardiac index; CTD, connective tissue disease; PAH, pulmonary arterial hypertension.

Of the patients with PAH, 13 (65%) had either the idiopathic or heritable form of the disease. New York Heart Association (NYHA) functional class was predominantly II (*n *= 15, 75%). RV function was normal, mildly, moderately, or severely dysfunctional in two (10%), six (32%), five (26%), and six (32%) of the patients, respectively. Right heart catheterization was performed a median (interquartile range) of 8 (5–17) months before the 6MWT and showed a right atrial pressure of 8.2 ± 5 mmHg, mean pulmonary artery pressure of 50.2 ± 19 mmHg, cardiac index of 2.8 ± 1 L/min/m^2^ and PVR: 10.4 ± 8 Wood units. All patients in the PAH group were receiving PAH‐specific therapies.

Patients with other lung diseases had either obstructive (*n *= 12, 71%) or restrictive disorders (5, 29%). Obstructive disorders included chronic obstructive pulmonary disease (COPD) (*n *= 8), asthma (*n *= 2), bronchiectasis (*n *= 1), and cystic fibrosis (*n *= 1); restrictive disorders encompassed interstitial lung disease (*n *= 4) and severe scoliosis (*n *= 1). Right ventricular function was mildly decreased in two (12%) patients, while in the rest it was normal. Right ventricular systolic pressure was estimated in 12 patients (29 ± 6 mmHg).

### Outcomes

During a median (IQR) follow‐up of 15 (13–27) months, seven (35%) patients with PAH had one or more events that were classified as clinical worsening (six patients required the initiation of new PAH‐specific treatments, three patients were hospitalized with right heart failure, one patient underwent lung transplantation, and one died). Of the patients with other lung diseases, four (24%) had clinical worsening including two that required hospitalizations for exacerbation of the respiratory condition, one patient was transplanted, and one died).

### Six‐minute walk test

#### Traditional determinations

Results of the traditional 6MWT measurements are shown in Table 2. Compared with healthy controls, patients with PAH and other lung diseases walked less, with similar heart rate at rest and during the test, but reduced heart rate recovery at 1, 2, and 3 min. When patients with PAH were compared with patients with other lung diseases the only significant differences occurred in HR recovery, which was slower in patients with other lung diseases.

**Table 2.** Traditional parameters measured during the 6‐min walk test (6MWT)

	PAH Mean ± SD or *n* (%)	Other lung diseases Mean ± SD or *n* (%)	Healthy controls Mean ± SD or *n* (%)	Comparison between PAH and other lung diseases (Wilcoxon–Mann–Whitney test or Fischer's exact test) *P* value
*n*	20	17	13	
Distance walked (m)	406 ± 113	397 ± 134	564 ± 69	0.86
Distance walked (% of predicted; Enright and Sherrill 1998)	67 ± 15	71 ± 23	97 ± 13	0.65
Need of O_2_ at baseline (%)	2 (10)	4 (24)	0 (0)	0.38
Need of O_2_ during walking (%)	8 (40)	8 (47)	0 (0)	0.75
Borg dyspnea scale	2.4 ± 2	2.7 ± 2	1.1 ± 1	0.78
Borg fatigue scale	2.4 ± 2	1.9 ± 2	1.1 ± 1	0.42
HR at baseline (bpm)	79 ± 11	81 ± 15	76 ± 14	0.39
Maximal HR (bpm)	127 ± 23	116 ± 20	126 ± 16	0.26
HR at 6 min (bpm)	120 ± 21	113 ± 20	126 ± 18	0.47
HR change (bpm)	48 ± 24	35 ± 13	49 ± 19	0.09
HR recovery at 1 min (bpm)	24 ± 11	16 ± 12	29 ± 11	0.02
HR recovery at 2 min (bpm)	33 ± 13	22 ± 12	39 ± 12	0.01
HR recovery at 3 min (bpm)	37 ± 15	27 ± 13	44 ± 13	0.03

bpm, beats per minute; HR, heart rate.

#### Beat‐by‐beat HR determinations during 6MWT activity and recovery among the three groups of subjects

**Figure 1.**
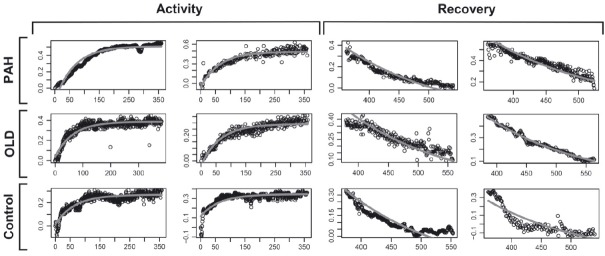
HR recordings during the activity and recovery phase of the 6‐min walk test (6MWT). Two patients in each group are presented. *X*‐axis shows the time in seconds and the *Y*‐axis the percentage change in HR. PAH, pulmonary arterial hypertension; OLD, other lung diseases.

**Figure 2.**
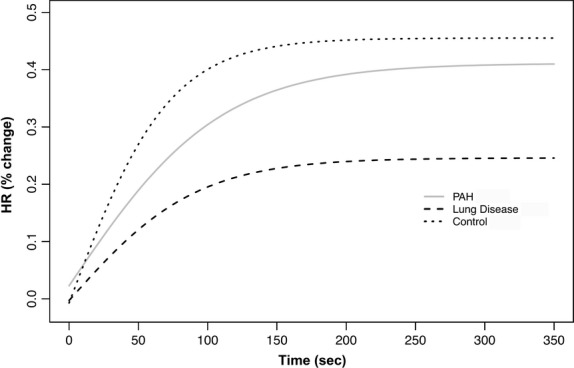
Nonlinear mixed model curves during the activity phase of the 6‐min walk test (6MWT). *X*‐axis shows the time in seconds and the *Y*‐axis shows the percentage change in HR from baseline. PAH, pulmonary arterial hypertension.

**Figure 3.**
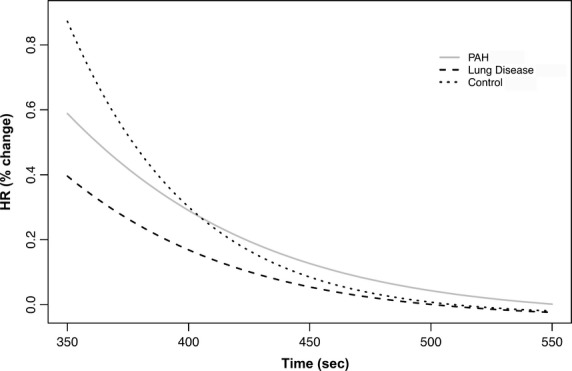
Nonlinear mixed model curves during the recovery phase of the 6‐min walk test (6MWT). *X*‐axis shows the time in seconds and the *Y*‐axis shows the percentage change in HR from the end of the walking phase. PAH, pulmonary arterial hypertension.

We recorded and average ± SD of 755 ± 191 HR determinations for each subjects. The beat‐by‐beat HR slopes were significantly different among all three groups of subjects either during the activity or recovery of the 6MWT. The parameter estimates during activity and recovery are presented in Tables 3 and 4, and the comparison between the estimates is shown in Tables 5 and 6, respectively. Heart rate data on two patients from each of the three groups are presented in Figure 1. Figure 2 presents the aggregated HR data showing a significant separation between the curves during activity, with a steeper HR increase in healthy controls that those with other lung diseases or PAH. Similarly, during recovery, the HR recovered more quickly in healthy controls than in patients with PAH or other lung diseases (Fig. 3). These results were not affected by the use of calcium channel blockers or beta‐blockers. In fact, the comparison between HR curves of patients with PAH and other lung diseases, both during the activity and recovery phases, varied significantly in at least one parameter with *P* values <0.001.

**Table 3.** Parameter estimates during the 6‐min walk test (6MWT)

Group	Parameter estimates				
	Parameter	Estimate	Standard error	DF	*P* value
PAH	*PAH1*	0.41	0.047	48	<0.0001
	*PAH2*	−0.36	0.068	48	<0.0001
	*PAH3*	0.14	0.001	48	<0.0001
OLD	*OLD1*	0.25	0.05	48	<0.0001
	*OLD2*	−0.26	0.07	48	0.001
	*OLD3*	0.11	0.002	48	<0.0001
Control	*C1*	0.45	0.06	48	<0.0001
	*C2*	−0.47	0.08	48	<0.0001
	*C3*	0.09	0.001	48	<0.0001
Random Effect	*RE1*	0.04	0.009	48	<0.0001
	*RE1*	−0.07	0.02	48	<0.0001
	*RE2*	0.19	0.04	48	<0.0001

Control, healthy controls; DF, degrees of freedom; PAH, pulmonary arterial hypertension; OLD, other lung diseases; RE, random effect.

**Table 4.** Parameter estimates during the recovery phase of the 6‐min walk test (6MWT)

Group	Parameter estimates				
	Parameter	Estimate	Standard error	DF	*P* value
PAH	*PAH4*	4.5	0.62	48	<0.0001
	*PAH5*	0.95	0.76	48	0.21
	*PAH6*	−0.17	0.003	48	<0.0001
OLD	*OLD4*	2.91	0.67	48	<0.0001
	*OLD5*	−0.38	0.84	48	0.66
	*OLD6*	−0.15	0.003	48	<0.0001
Control	*C4*	9.26	0.82	48	<0.0001
	*C5*	−0.22	0.97	48	0.82
	*C6*	−0.12	0.002	48	<0.0001
Random effect	*RE4*	7.42	1.04	48	<0.0001
	*RE5*	−2.08	0.21	48	<0.0001
	*RE6*	8.89	1.12	48	<0.0001

Control, healthy controls; DF, degrees of freedom; PAH, pulmonary arterial hypertension; OLD, other lung diseases; RE, random effect.

**Table 5.** Comparison among the groups of parameter estimates during the 6‐min walk test (6MWT)

Comparison	Additional estimates				
	Label	Estimate	Standard error	DF	*P* value
PAH ‐ OLD	PAH1‐OLD1	0.16	0.07	48	0.02
	PAH1‐OLD2	−0.11	0.1	48	0.29
	PAH3‐OLD3	0.02	0.002	48	<0.0001
PAH ‐ Control	PAH1‐C1	−0.04	0.07	48	0.57
	PAH2‐C2	0.11	0.11	48	0.32
	PAH3‐C3	0.05	0.002	48	<0.0001
Control ‐ OLD	C1‐OLD1	0.2	0.08	48	0.01
	C2‐OLD2	−0.21	0.11	48	0.06
	C3‐OLD3	−0.02	0.002	48	<0.0001

Control, healthy controls; DF, degrees of freedom; PAH, pulmonary arterial hypertension; OLD, other lung diseases.

**Table 6.** Comparison among the groups of parameter estimates during the recovery phase of the 6‐min walk test (6MWT)

Comparison	Additional estimates				
	Label	Estimate	standard error	DF	Pr > |t|
PAH ‐ OLD	PAH4‐OLD4	1.59	0.92	48	0.09
	PAH5‐OLD5	1.33	1.12	48	0.24
	PAH6‐OLD6	−0.02	0.005	48	0.0009
PAH ‐ Control	PAH4‐C4	−4.76	1.03	48	<0.0001
	PAH5‐C5	1.17	1.22	48	0.34
	PAH6‐C6	−0.05	0.003	48	<0.0001
Control ‐ OLD	C4‐OLD4	6.35	1.06	48	<0.0001
	C5‐OLD5	0.16	1.28	48	0.9
	C6‐OLD6	0.03	0.004	48	<0.0001

Control, healthy controls; DF, degrees of freedom; PAH, pulmonary arterial hypertension; OLD, other lung diseases.

#### Beat‐by‐beat HR determinations between patients who had or not progression of the disease

**Figure 4.**
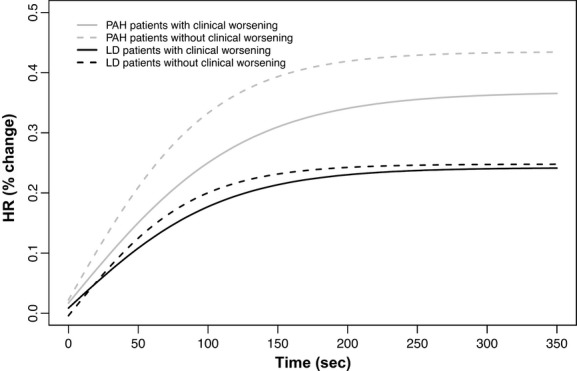
Nonlinear mixed model curves during the activity phase of the 6‐min walk test (6MWT), contrasting patients with and without clinical worsening. *X*‐axis shows the time in seconds and the *Y*‐axis shows the percentage change in HR from baseline. PAH, pulmonary arterial hypertension.

During the activity phase of the 6MWT, the HR slope of PAH patients with clinical worsening (*n *= 7) revealed a slower rate of change than the one obtained in PAH patients without clinical worsening (*n *= 13; Fig. 4). The distance walked in meters (415 ± 150 vs. 401 ± 95 m, *P* = 0.91) or percentage of predicted 6‐min walk distance (69 ± 21 vs. 66 ± 12%, *P* = 0.97) and HRR at 1 (28 ± 10 vs. 22 ± 11 bpm, *P* = 0.18) min were not statistically different between the patients with and without progression of disease.

Similarly, the HR slopes during the 6MWT of patients with other lung diseases who had progression of the disease (*n *= 4) were significantly different (less steep) than the ones that did not (Fig. 4). Differences in walk distance were not statistically different; however, the HRR at 1 min was significantly slower in patients who had progression of the disease (7 ± 5 vs. 19 ± 12 bpm, *P* = 0.02).

**Figure 5.**
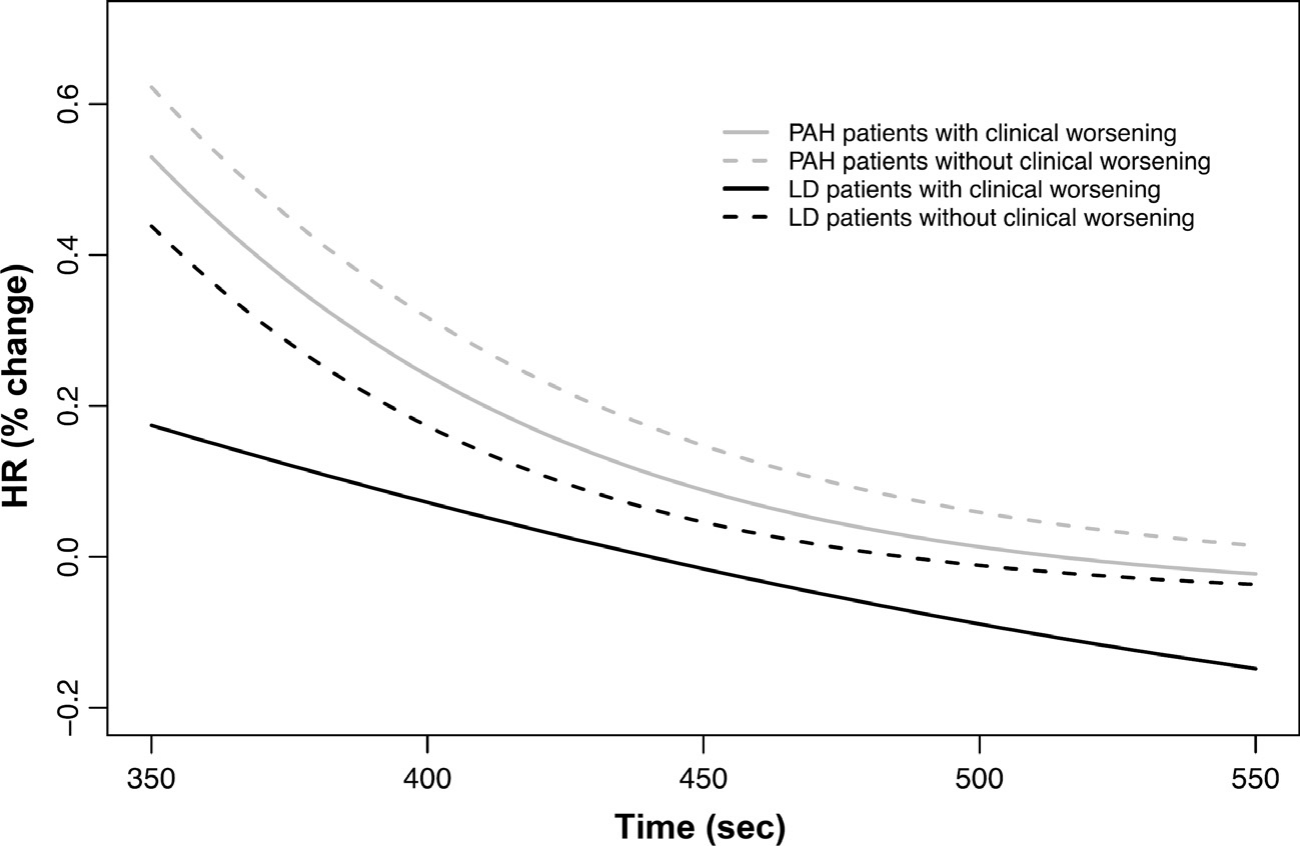
Nonlinear mixed model curves during the recovery phase of the 6‐min walk test (6MWT), contrasting patients with and without clinical worsening. *X*‐axis shows the time in seconds and the *Y*‐axis shows the percentage change in HR from the end of the walking phase. PAH, pulmonary arterial hypertension.

Interestingly, during the recovery phase of the 6MWT, there was no statistical difference between the HR curves of patients with PAH who had or not progression of their disease (Fig. 5). However, the HR slopes during 6MWT recovery was statistically different in patients with other lung diseases who experienced progression of the disease compared to the ones that did not (Fig. 5).

## Discussion

In this study, we contrasted the beat‐by‐beat HR acceleration and recovery slopes during 6MWT in patients with PAH, other lung diseases, and healthy controls. Upon analyzing a large number of HR determinations using modern statistical methodologies, we showed that patients with PAH had less pronounced HR slopes, both during the activity and recovery phases of the 6MWT, when compared to the other groups. More importantly, the HR acceleration rate was more pronounced in patients with PAH who did not experience clinical worsening, compared to those that progressed.

Recent advances in technology permitted the development of a portable, signal‐morphology‐based impedance cardiograph that provides wireless real‐time measures of a variety of hemodynamic parameters as well as HR. We have previously shown that it is possible to incorporate this novel and noninvasive technology as part of the traditional 6MWT (Tonelli et al. 2013). Portable heart rate monitors that accurately record the RR intervals permit a clear identification of the different phases of the 6MWT, that is rest, activity, and recovery periods, and present the results in an electronic format would be likewise useful to analyze the HR acceleration and deceleration slopes.

The increasing metabolic demands during 6MWT requires an increase in HR, an effect that is achieved by enhancing the sympathetic and decreasing the parasympathetic regulation (Arai et al. 1989). The reverse occurs when the exercise is stopped, that is, the sympathetic stimulation is withdrawn and the parasympathetic regulation is reinstated (Savin et al. 1982; Imai et al. 1994; Kannankeril et al. 2004). We noted slower chronotropic responses during activity and recovery in patients compared to controls. Similarly, the chronotropic response during activity and recovery is altered in a variety of diseases as a result of autonomic imbalance. Diseases like congestive heart failure or PAH have an increased basal sympathetic activity that results in a higher resting HR but lower peak heart rate with a slower sympathetic response time due to downregulation of beta receptors (Colucci et al. 1989; Mak et al. 2012; Pierpont et al. 2013). In addition, as the vagal tone is also decreased in these patients, the HRR is blunted (Imai et al. 1994). Interestingly, the kinetics of change in oxygen consumption (quantified by mean response time) during exercise and recovery are impaired in pulmonary PAH in part due to slowed heart rate kinetics in this disease (Riley et al. 2000).

In our study, a slower chronotropic response during the activity phase of 6MWT was associated with clinical worsening. Likewise, an attenuated HR response to exercise and a delayed HRR are predictors of all‐cause mortality in a variety of conditions (Lauer et al. 1996, 1999; Chaitman 2003; Maddox et al. 2008). The mechanisms for this association remain unclear but may reflect a different modulation of the autonomic tone in patients with more severe cardiovascular conditions (Lauer et al. 1999), sinus node remodeling (Pierpont et al. 2013), enhanced systemic inflammation, and endothelial vasodilator dysfunction (Huang et al. 2006). In fact, an increase in the sympathetic tone may be associated with endothelial dysfunction (Hijmering et al. 2002).

Interestingly, other studies observed that autonomic dysfunction, measured by HR variability and baroreflex sensitivity, was associated with diminished exercise capacity and clinical deterioration in patients with PAH (Wensel et al. 2009; Ciarka et al. 2010). Delayed HRR predicts clinical worsening and mortality in PAH (Dimopoulos et al. 2009; Minai et al. 2012; Ramos et al. 2012). Furthermore, patients with PAH commonly have a blunted chronotropic response that relates to disease severity or chronic illness deconditioning (Provencher et al. 2006; Dimopoulos et al. 2009). Similarly, autonomic dysfunction has been described in patients with COPD based on a decreased HR variability, decrease HR response to standing, and abnormal cardiac sympathetic nervous function (Stewart et al. 1991; Volterrani et al. 1994; Sakamaki et al. 1999). In fact, patients with COPD commonly have impaired chronotropic response and delayed HRR, a prevalence that increases with disease severity and likely reflects autonomic dysfunction (Lacasse et al. 2005; Gupta et al. 2013).

The differences in exertion levels among the group of subjects in this study may be a factor that could explain the dissimilar HR slopes observed. However, the distance walked in patients with PAH and other lung diseases were similar, despite significant differences in the HR slopes during 6MWT activity and recovery. Furthermore, the distance walked was also similar between the subgroups of patients with PAH or other lung diseases who did or did not experience clinical worsening. The slower HR acceleration and deceleration observed in patients with PAH or those with disease progression might be explained by a greater degree of autonomic dysfunction and/or sinus node remodeling in this condition; however, this requires further study.

Our study has limitations: (1) PH was considered not present in patients with other lung diseases based on echocardiography, a methodology that has its limitations and could lead to misclassifications, (2) most of the PH patients were receiving PH‐specific treatment; thus, the results observed in this group of patients are not applicable to treatment naïve individuals, (3) we included a relatively small number of patients in this preliminary study; however, this time series with high‐frequency data points (>600 HR determination per subject) increased the statistical power to detect differences between the groups and (4) the group of patients with other lung diseases is heterogeneous but it constitutes an interesting group to compare the findings noted in PH patients. In spite of these limitations, our study is the first to demonstrate that there are differences in the HR slopes during 6MWT between patients with pulmonary vascular or other lung diseases and control. Remarkable is the fact that PAH that experience clinical worsening during the follow‐up had slower HR acceleration during the activity phase of the 6MWT. Future research will test whether HR slopes change in PAH patients after the initiation of PAH‐specific therapies.

## Conclusions

The HR slopes during 6‐min walk and subsequent recovery are steeper in healthy controls than patients with PAH or other lung diseases. Patients who had progression of PAH or other lung diseases had slower HR acceleration curves than those without deterioration of their medical condition.

## Acknowledgments

We appreciate the invaluable help provided by the pulmonary function test laboratory personnel at Cleveland Clinic who made this study possible. We are thankful to Manatec Biomedical for providing us with PhysioFlow Enduro and technical assistance.

## Conflict of Interest

Adriano R. Tonelli MD, Laith Alkukhun MD, Xiao‐feng Wang PhD, Qi Zhang MS, and Raed A. Dweik MD: The author has no significant conflicts of interest with any companies or organization whose products or services may be discussed in this article. Omar A. Minai MD: Member Scientific Advisory Board: Actelion, Gilead, and Bayer. Member Speakers Bureau: Actelion and Gilead.
